# EphA2 as a phase separation protein associated with ferroptosis and immune cell infiltration in colorectal cancer

**DOI:** 10.18632/aging.205212

**Published:** 2023-11-16

**Authors:** Yanling Li, Qiu Peng, Lujuan Wang

**Affiliations:** 1 Hunan Cancer Hospital and The Affiliated Cancer Hospital of Xiangya School of Medicine, Central South University, Changsha 410013, Hunan, China; 2 Hunan Key Laboratory of Tumor Models and Individualized Medicine, The Second Xiangya Hospital of Central South University, Changsha 410011, Hunan, China

**Keywords:** colorectal cancer, EphA2, liquid-liquid phase separation, ferroptosis, immune cell infiltration

## Abstract

Colorectal cancer is one of the most common malignant tumors in the digestive system, and its high incidence and metastasis rate make it a terrible killer that threatens human health. In-depth exploration of the targets affecting the progression of colorectal cancer cells and the development of specific targeted drugs for them are of great significance for the prognosis of colorectal cancer patients. Erythropoietin-producing hepatocellular A2 (EphA2) is a member of the Eph subfamily with tyrosine kinase activity, plays a key role in the regulation of signaling pathways related to the malignant phenotype of various tumor cells, but its specific regulatory mechanism in colorectal cancer needs to be further clarified. Here, we found that EphA2 was abnormally highly expressed in colorectal cancer and that patients with colorectal cancer with high EphA2 expression had a worse prognosis. We also found that EphA2 can form liquid-liquid phase separation condensates on cell membrane, which can be disrupted by ALW-II-41-27, an inhibitor of EphA2. In addition, we found that EphA2 expression in colorectal cancer was positively correlated with the expression of ferroptosis-related genes and the infiltration of multiple immune cells. These findings suggest that EphA2 is a novel membrane protein with phase separation ability and is associated with ferroptosis and immune cell infiltration, which further suggests that malignant progression of colorectal cancer may be inhibited by suppressing the phase separation ability of EphA2.

## INTRODUCTION

Colorectal cancer is the third most prevalent malignancy and the second most deadly, and is a major public health problem worldwide. It is a major public health problem worldwide [1, 2]. Surgery is the treatment of choice for primary colorectal cancer and has the advantages of being effective and non-biologically resistant, but it is risky and traumatic. Radiotherapy and chemotherapy are often used as adjuvant therapies to surgical treatment, but have high toxic side effects. With the improvement of diagnosis and advances in targeted therapy technology, there is an increasing interest in molecular targets for colorectal cancer that indicate significant effects [3–5].

The Eph receptor family is the largest family of receptor tyrosine kinases and is a key regulator of cell growth, differentiation and motility [6–8]. Eph receptors and their ligands, Ephrin proteins (Eph receptor interacting proteins), play a key role in many pathological states (abnormally elevated RTK activity is a feature of most human cancers), and therefore Eph receptors can be used as potential drug targets [9, 10]. There are 14 Eph receptors in the human genome, which can be subdivided into EphA and EphB subclasses. EphA2 is a member of the Eph family, and high expression of EphA2 occurs in a variety of tumors, such as liver cancer, breast cancer, colorectal cancers, bladder cancer and glioblastoma, and EphA2 is a key driver of metastasis and a predictor of poor prognosis in several tumors [9, 11–13]. EphA2 has both classical and non-classical modes of driving tumorigenesis. The classical mode means that EphA2 inhibits positive signaling of ligands and tyrosine kinases, thereby suppressing tumorigenesis. Inhibiting Focal Adhesion Kinase (FAK), Protein Kinase B (PKB) and Extracellular Regulated Rotein Kinases (ERK) affects cell motility and survival. The non-classical pathway refers to EphA2 ligands and tyrosine kinase non-dependent activation and phosphorylation. Inflammatory cytokines and growth factors via RSK AKT and Protein Kinase A (PKA) induce phosphorylation of EphA2 Ser897. This phosphorylation is able to localize EphA2 at the frontier of migrating cells, leading to actin cytoskeleton framework assembly and lamellipid membrane formation, promoting and maintaining certain cancer cell functions, such as cell motility and proliferation [14, 15].

In recent years, phase separation of biological macromolecules has gained much attention. Phase separation, also known as biomolecular condensates, is involved in regulating a variety of physiological processes, including gene expression, DNA damage repair, signal transduction, cellular metabolism, and immune regulation, by forming membraneless organelles [15–20]. Multivalent interactions are key in driving phase separation [21, 22]. Dysregulation of phase separation can lead to the formation of abnormal condensates, which can cause a variety of human diseases, such as cancer and neurodegenerative diseases [23, 24]. More and more studies are now also focusing on cell membrane molecules that can also transmit cellular signals through the occurrence of phase separation, including T cell receptors, androgen receptors, receptor tyrosine kinases, etc., [25–27]. Whether EphA2, an important receptor tyrosine kinase, can promote tumor development through the occurrence of phase separation has not been reported.

Here, we found that EphA2 was abnormally highly expressed in colorectal cancer tissues, and patients with high EphA2 expression had a worse prognosis. Interestingly, we found that EphA2 has multiple intrinsically disordered regions (IDRs), which is an important basis for the occurrence of phase separation [28, 29]. Indeed, we also further confirmed in the cells that EphA2 can undergo phase separation at the cell membrane. The EphA2 phase separation was significantly disrupted when treated with ALW-II-41-27, an inhibitor of EphA2. In addition, we also found that EphA2 expression was significantly correlated with the expression of ferroptosis-related genes and the infiltration of immune cells. These results suggest that EphA2 may perform its tumor-promoting function through its phase-separating properties, which also provides a possibility to treat tumors by developing drugs that target EphA2 phase separation.

## MATERIALS AND METHODS

### Expression analysis

UALCAN (http://ualcan.path.uab.edu/) is a comprehensive website for analyzing canceromics data. We used UALCAN to analyze mRNA and protein expression levels of EphA2 in colorectal cancer samples and normal samples from The Cancer Genome Atlas (TCGA) and Clinical Proteomic Tumor Analysis Consortium (CPTAC) databases.

We analyzed the mRNA expression levels of EphA2 in colorectal cancer tissues and normal tissues using two datasets numbered GSE21815 and GSE37182 from the Gene Expression Omnibus (GEO) database.

### Immunohistochemical analysis (IHC)

The Human Protein Atlas database (HPA) (http://www.proteinatlas.org) contains protein expression images in various cells, tissues and organs. We analyzed the immunohistochemical images of EphA2 in colorectal cancer tissues and normal tissues using HPA database.

### Survival analysis

The Kaplan Meier plotter database (http://kmplot.com/analysis/) is able to assess the correlation between gene expression and survival in a variety of tumor types. We used this database to analyze the correlation between EphA2 expression and patient survival in colorectal cancer patients.

### Tumor mutational burden (TMB) and microsatellite instability (MSI) analysis

Gene expression data from the TCGA database for colorectal cancer and the corresponding clinical information were used. The correlation of EphA2 expression with TMB and MSI was examined using Spearman's correlation analysis.

### Analysis of internal disordered regions (IDRs) and net charge per residue (NCPR) of proteins

The amino acid sequence of EphA2 was uploaded to the Predictor of Natural Disordered Regions (PONDR) database (http://www.pondr.com) to analyze its internal disordered region. The Classification of Intrinsically Disordered Ensemble Regions (CIDER) database (http://157.245.85.131:8000/CIDER/) allows the calculation of many different parameters associated with disordered protein sequences. We used this database to analyze the net charge per residue of EphA2 amino acid sequence.

### Live cell immunofluorescence assay

The cells were incubated in an incubator for approximately 6 hours and then transfected with the EGFP-EphA2 plasmid when the cells were sufficiently attached to the wall. Leica TCS SP8 confocal microscope was used for imaging. Cells were imaged on a heated plate (37°C) at all times during the imaging process.

### Fluorescence recovery after photobleaching (FRAP) assay

FRAP was performed using a Leica TCS SP8 confocal microscope and 2.4 mW laser intensity for bleaching at room temperature, and a 63 × /1.4 oil immersion objective and photomultiplier detector for imaging. Considering the size of the droplets, different droplets and target areas were selected for the FRAP experiments in cells, and in each FRAP experiment, another region with the same fluorescence intensity as the droplet size used for photobleaching was recorded for fluorescence intensity correction.

## RESULTS

### EphA2 is aberrantly highly expressed in colorectal cancer and is associated with prognosis

**Figure 1:**
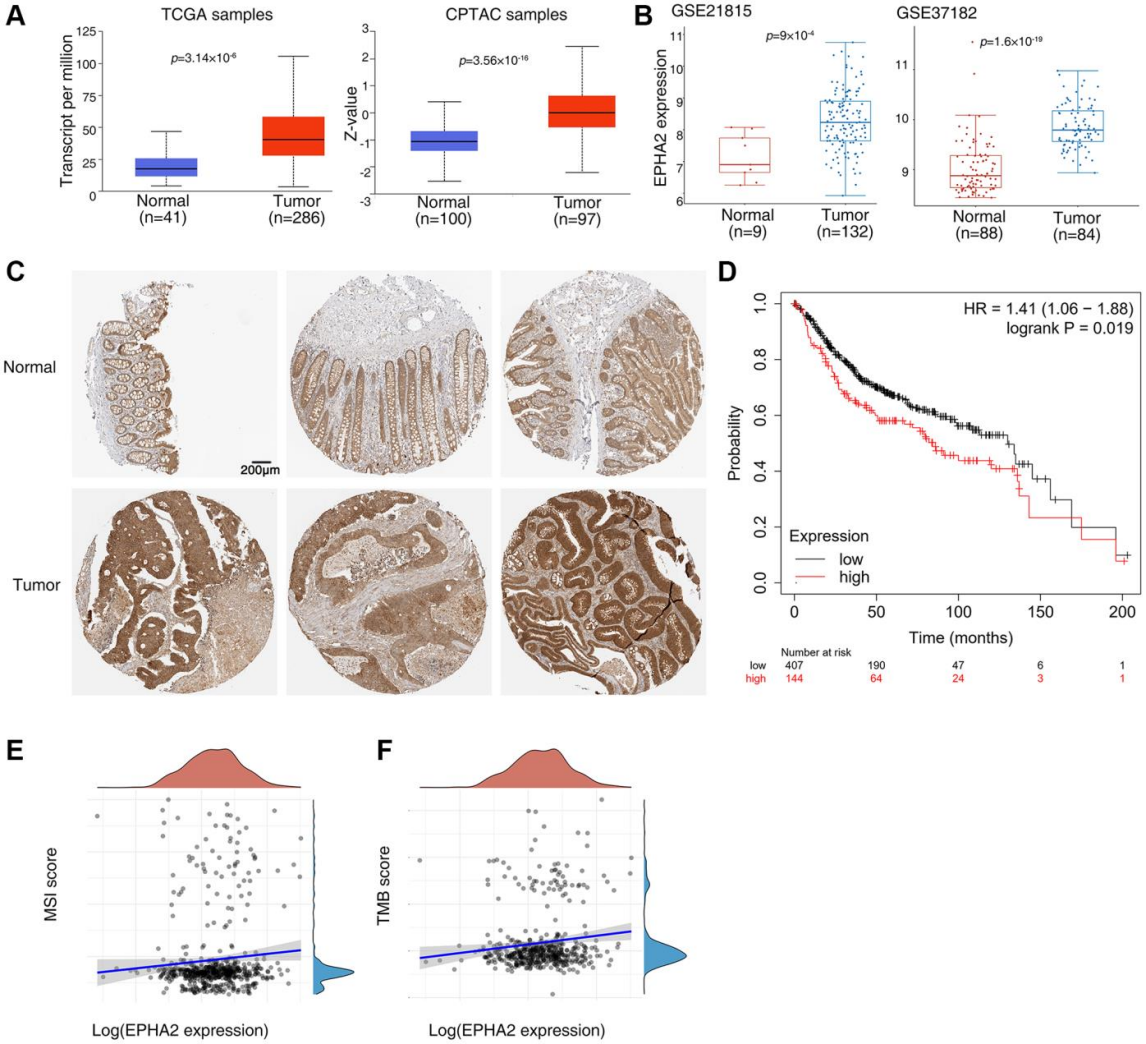
**Abnormal expression of EphA2 in colorectal cancer and its relationship with prognosis.** (**A**) Analysis of EphA2 mRNA and protein expression in colorectal cancer tissues and normal tissues using the UALCAN database. (**B**) Analysis of EphA2 mRNA expression in colorectal cancer tissues and normal tissues using the GEO database. (**C**) EphA2 expression data from HPA database in colorectal cancer tissues and normal tissues. (**D**) Overall survival of colorectal patients with differential EphA2 expression. (**E**, **F**) Correlation of EphA2 expression with MSI and TMB in colorectal cancer patients.

To verify the correlation between EphA2 and colorectal cancer, we found that the mRNA expression level of EphA2 was significantly higher in colorectal samples than in normal tissues using TCGA samples from the UALCAN database (https://ualcan.path.uab.edu/index.html), and similarly, we analyzed the protein expression level of EphA2 using CPTAC samples and also found that its expression was significantly higher in colorectal cancer tissues than in normal samples (Figure 1A). We further analyzed two datasets in the GEO database (GSE21815 and GSE37182) and found that EphA2 expression was significantly upregulated in tumors (Figure 1B). Then, we assessed the protein expression levels of EphA2 in normal and colorectal cancer tissues by tissue samples from the Human Protein Atlas database. The immunohistochemical (IHC) staining data as shown in Figure 1C revealed that the protein expression level of EphA2 was significantly elevated in tumor tissues. Using the Kaplan Meier plotter database (http://kmplot.com/analysis/), we analysed the relevance of EphA2 expression to the prognosis of colorectal cancer patients and found that among 551 colorectal cancer patients, those with high EphA2 expression had a worse prognosis (Figure 1D). MSI and TMB have been used as predictive biomarkers for immunotherapy in a variety of tumours [30–32]. We analysed the correlation between EphA2 expression and MSI and TMB using TCGA colorectal cancer data and found a significant positive correlation between EphA2 expression and MSI and TMB scores (Figure 1E, 1F).

### EphA2 shows properties of liquid–liquid phase-separated condensates

Phase separation is the main mechanism for the formation of membraneless compartments in cells and is involved in the regulation of cellular metabolism, signal transduction, gene expression and protein homeostasis. In recent years, researchers have found that phase separation and cancer are closely linked, by affecting DNA repair processes, transcriptional regulation as well as the assembly of important membrane-free compartments in cancer [23, 33–35].

**Figure 2:**
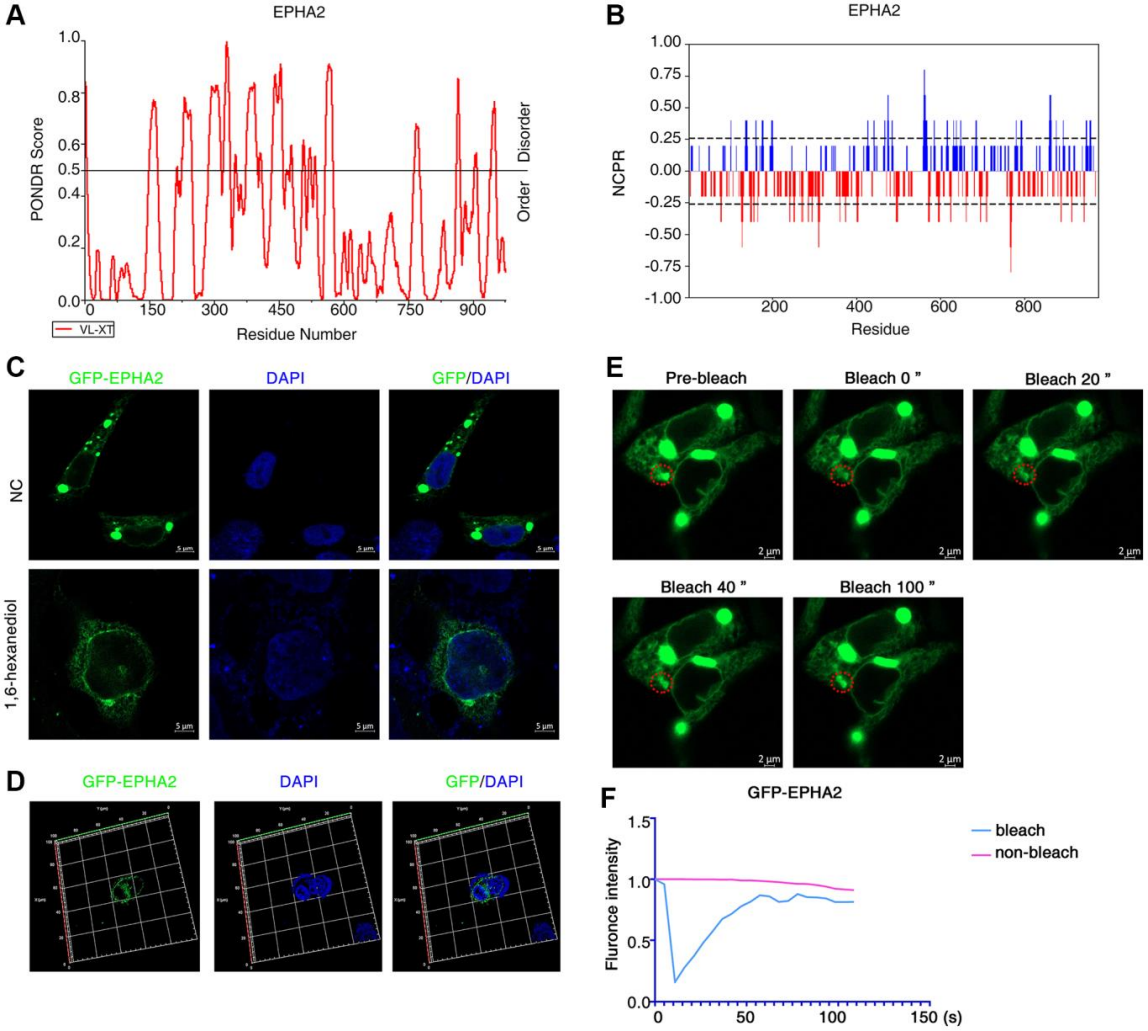
**EphA2 can undergo phase separation in cells.** (**A**, **B**) Predictions of IDRs and NCPR of EphA2 using PONDR and CIDER database based on their amino acid positions and sequences. (**C**) Transfection of GFP-EphA2 in HEK293 cells and immunofluorescence detection of EphA2 puncta in the presence or absence of 1,6 hexanediol treatment. (**D**) Transfection of GFP-EphA2 in HEK293 cells and detection of EphA2 puncta by immunofluorescence 3D imaging. (**E**) Representative images of FRAP experiment of HEK293 cells that had been transfected with GFP-EphA2 expression vector. The red circle highlights the puncta undergoing targeted bleaching. (**F**) Quantification of FRAP fluorescence intensity data for GFP-EphA2 puncta.

**Figure 3:**
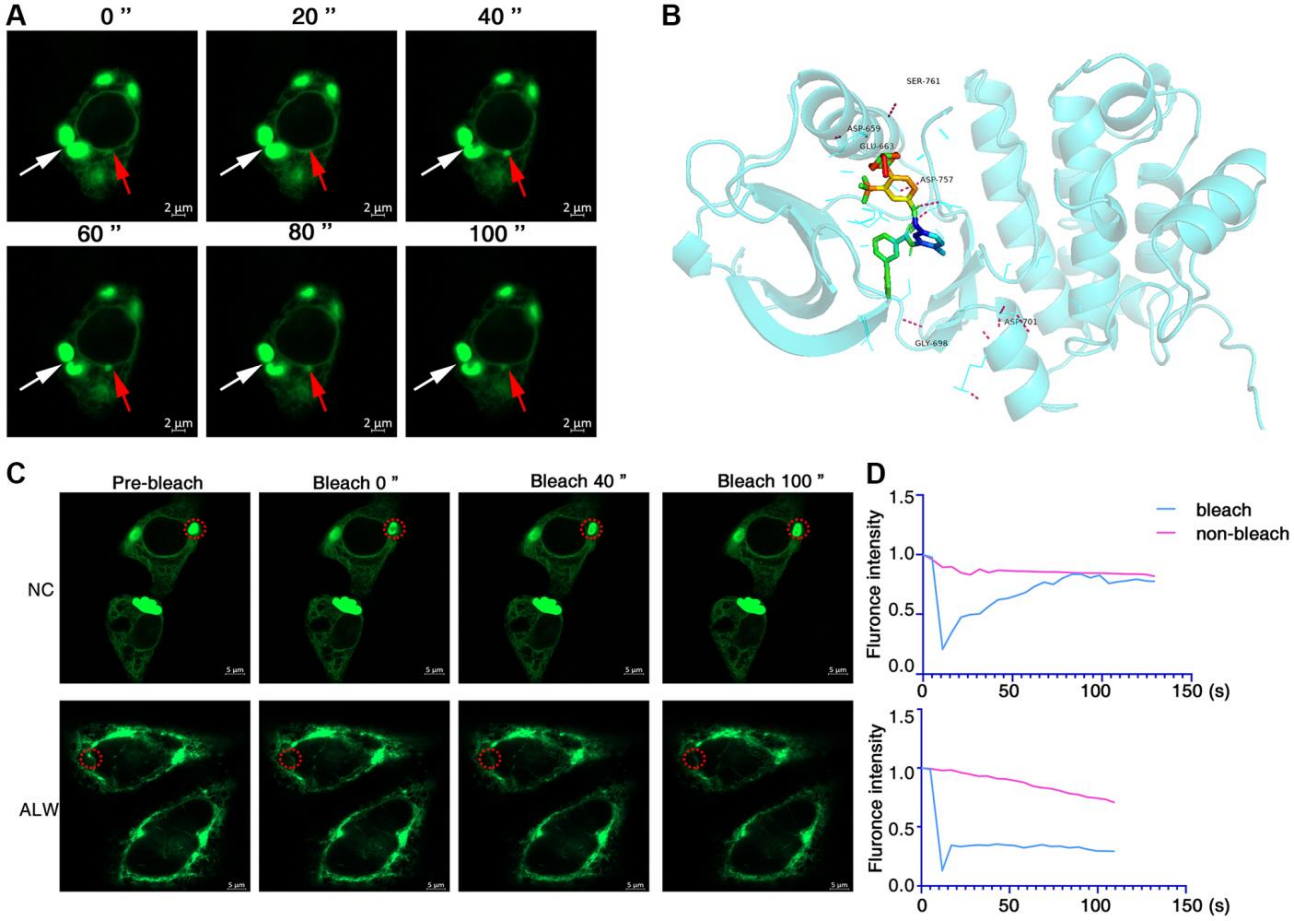
**ALW can disrupt EphA2 phase separation condensates.** (**A**) Immunofluorescence observation of the dynamics of GFP-EphA2 condensates in living cells. The red arrow highlights the dynamic process of GFP-EphA2 condensates at different times. (**B**) Molecular docking showing the binding site of ALW to EphA2. (**C**) Transfection of GFP-EphA2 in HEK293 cells and FRAP experiment detection of EphA2 puncta in the presence or absence of ALW treatment. (**D**) Quantification of FRAP fluorescence intensity data for GFP-EphA2 puncta.

To investigate whether EphA2 can undergo phase separation, we first analysed the internal disordered region and net charge per residue of EphA2 amino acid, which are important factors for phase separation to occur [36–38], using the PONDR and CIDER databases. We found that EphA2 has a significant internal disordered region (IDR) and that its amino acid residues carry a significant net charge (Figure 2A, 2B). To further confirm that EphA2 can undergo phase separation, we transfected GFP-EphA2 fusion plasmids into HEK293 cells and used immunofluorescence assays to find that EphA2 forms puncta on the cell membrane, which may be condensates formed by phase separation. We further found that EphA2 puncta were significantly disrupted after treatment of GFP-EphA2-expressing cells with the phase separation disruption reagent 1,6 hexanediol (Figure 2C, 2D). An important feature of phase-separated condensates is that they exhibit rapid exchange kinetics with their surroundings [39, 40]. To demonstrate the dynamic characteristics of GFP-EphA2 puncta, we performed fluorescence recovery after photobleaching (FRAP) experiments in GFP-EphA2-positive live cells and found that bleaching the GFP-EphA2 puncta could be followed by rapid recovery within a short period of time (Figure 2E, 2F). Furthermore, we found that individual GFP-EphA2 condensates exhibit rapid changes in size and fluorescence intensity, and that when the condensates meet each other they aggregate to form larger puncta (Figure 3A). These results suggest that EphA2 forms punctate structures on cell membranes with properties consistent with liquid-liquid phase separation condensates.

### Inhibitors of EphA2 can disrupt its phase separation properties

The ATP-competitive tyrosine kinase inhibitor ALW-II-41-27 (ALW) of EphA2 has been shown to inhibit the growth of a variety of tumour cells *in vitro* and *in vivo* [9, 41]. To investigate the effect of ALW on EphA2 phase separation, we first used molecular docking techniques to demonstrate that ALW can bind to multiple pockets of EphA2 protein (Figure 3B). We also further found, using FRAP experiments, that the dynamic properties of the bleached GFP-EphA2 phase separation condensate were significantly weaker after treating the cells with ALW (Figure 3C, 3D). These results suggest that inhibitors of EphA2 can disrupt its phase separation properties.

### EphA2 is associated with ferroptosis

**Figure 4:**
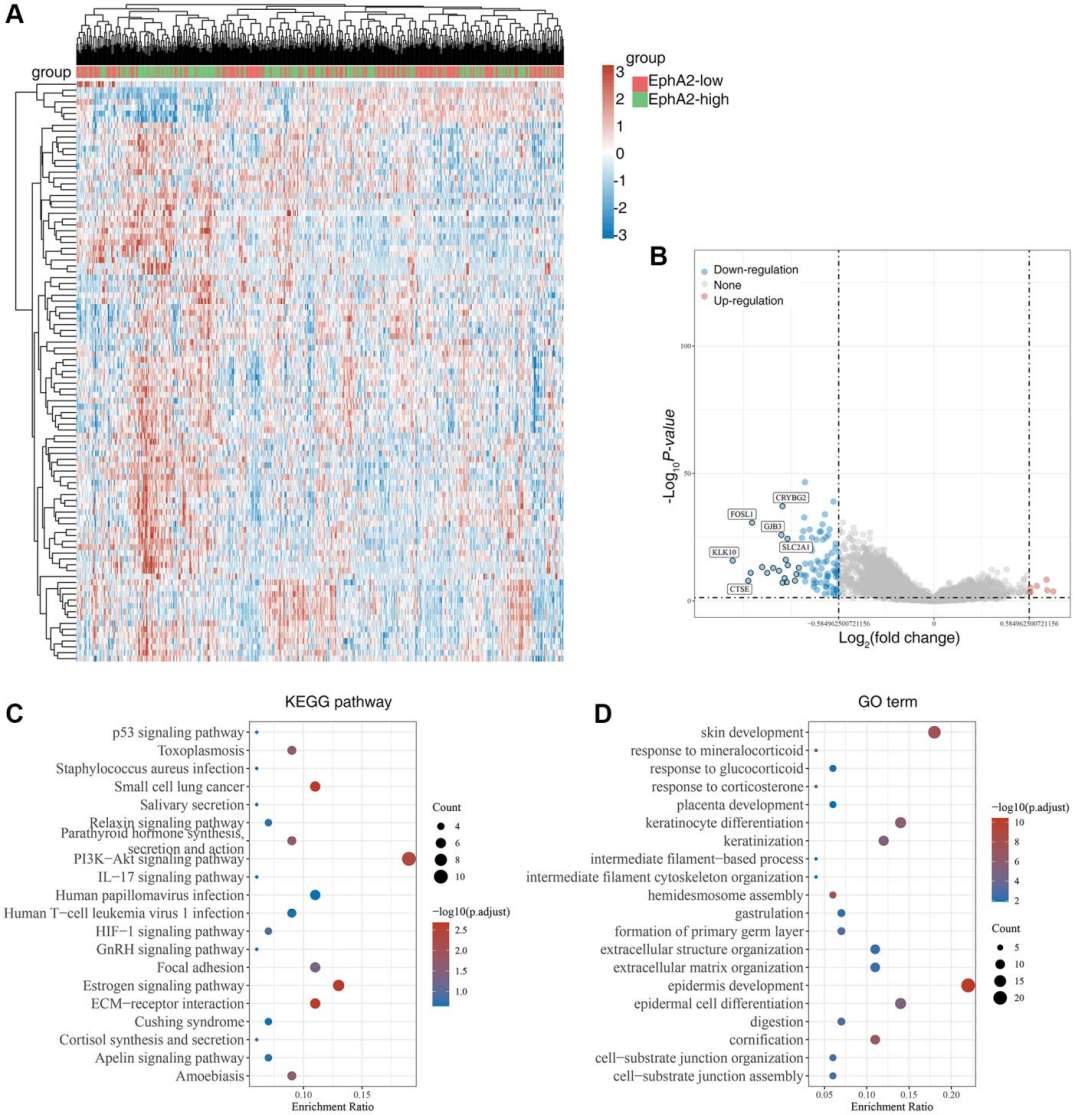
**Biological functions of EphA2 in colorectal cancer samples.** (**A**) Heat map showing differentially expressed genes in colorectal cancers with high and low expression of EphA2. (**B**) Venn diagram showing EphA2-regulated differentially expressed genes. (**C**, **D**) KEGG and GO analyses of EphA2-regulated differentially expressed genes in colorectal cancers patients.

**Figure 5:**
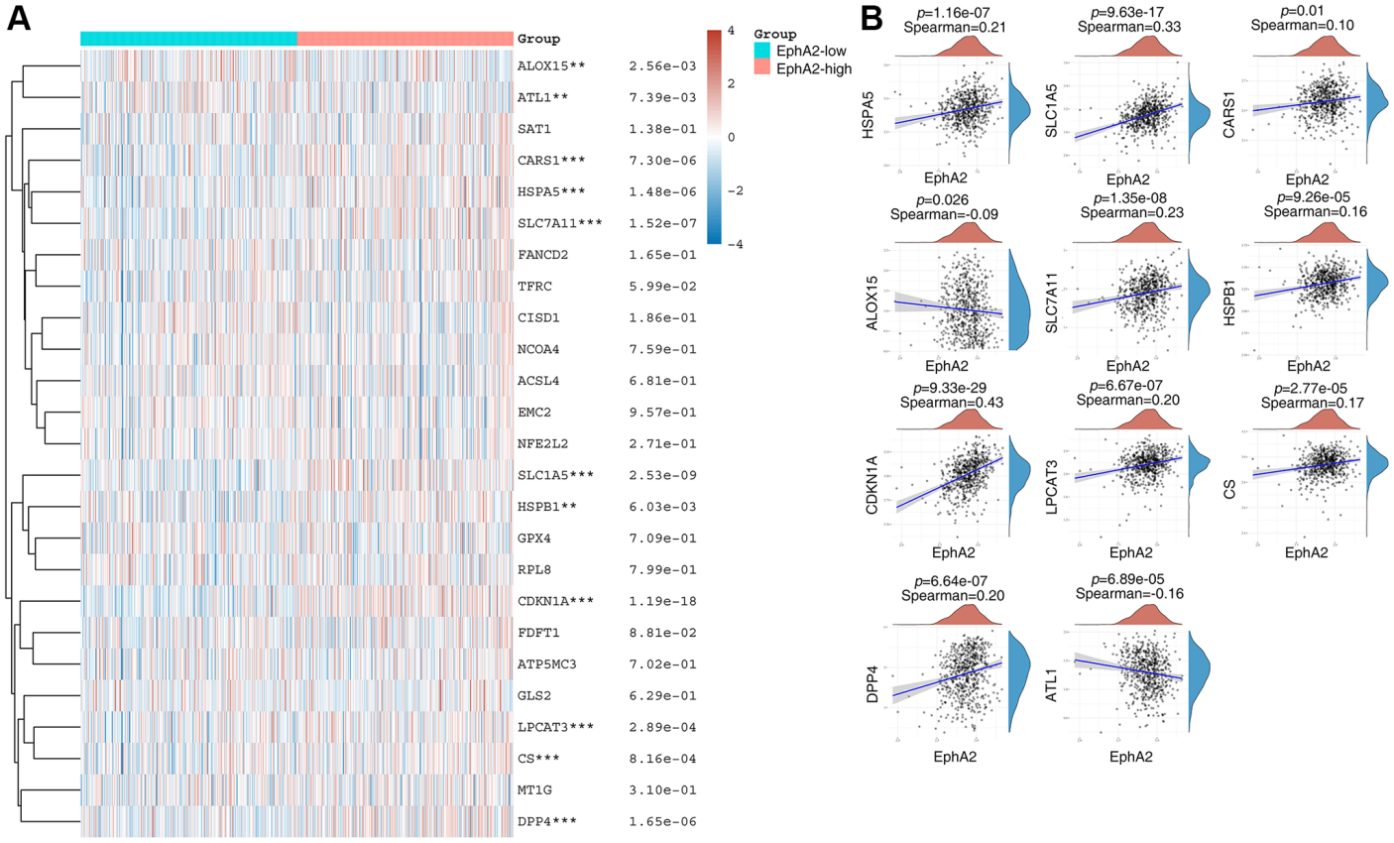
**EphA2 is associated with ferroptosis.** (**A**) Heat map showing differential expression of ferroptosis-related genes in EphA2 differentially expressed colorectal cancer patients. (**B**) Correlation of EphA2 expression with the expression of common ferroptosis-related genes. ^*^stands for significance levels, ^*^ for *p* < 0.05, ^**^p < 0.01, ^***^p < 0.001.

Ferroptosis is a form of programmed cell death different from apoptosis and necrosis, characterized by the accumulation of peroxidized lipids in the cell membrane and intracellular reactive oxygen species clusters up to lethal levels, mainly involving biological processes such as glutathione metabolism, iron metabolism, lipid metabolism and oxidative stress. In recent years, numerous studies have identified the role of ferroptosis in anti-tumour, and targeting ferroptosis-related mechanism pathways can effectively inhibit tumourigenesis and development [42–44]. To explore the relationship between EphA2 and ferroptosis, we first analysed differential gene expression in samples with differential EphA2 expression using TCGA colorectal cancer samples, and in total we identified seven up-regulated and 92 down-regulated genes (Figure 4A, 4B). We further performed KEGG and GO analysis on the differential genes regulated by EphA2 and found that these gene-enriched pathways are closely related to tumor development, such as p53, HIF-1, PI3K and other signaling pathways (Figure 4C, 4D). Furthermore, we analyzed the correlation between EphA2 expression and the expression of common ferroptosis-related genes in colorectal cancer and found that EphA2 expression was significantly correlated with the expression of several ferroptosis-related genes (Figure 5A, 5B). These results suggest that EphA2 may influence the development of colorectal cancer through the regulation of ferroptosis.

### EphA2 is associated with immune cell infiltration

**Figure 6:**
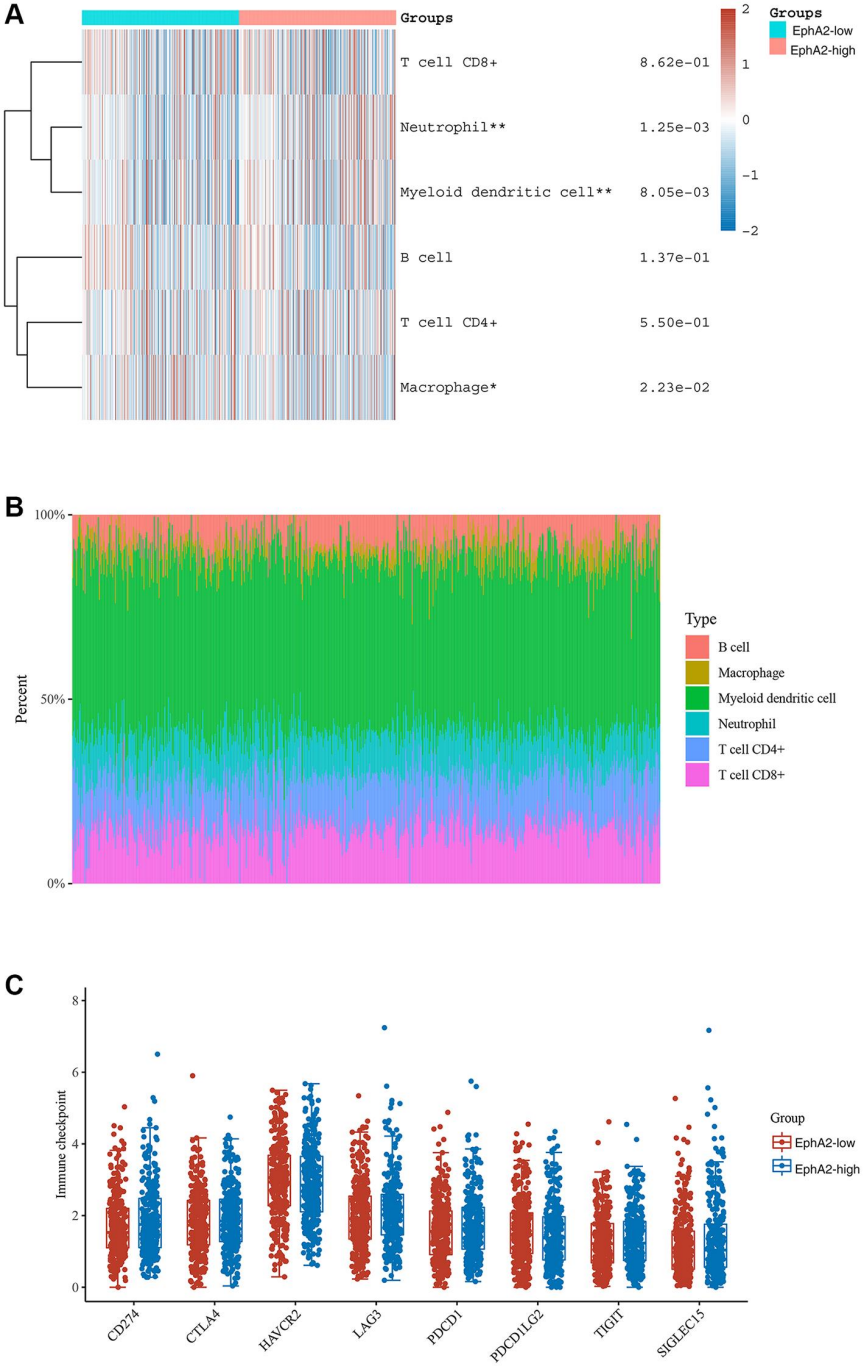
**Correlation between EphA2 expression and various immune cells infiltration of colorectal cancer.** (**A**) Heat map showing the level of immune cell infiltration in EphA2 differentially expressed colorectal cancer patients. (**B**) Percentage abundance of tumour-infiltrating immune cells per sample. (**C**) Expression distribution of immune checkpoint of colorectal cancer samples with differential EphA2 expression. ^*^stands for significance levels, ^*^ for *p* < 0.05, ^**^p < 0.01.

Tumour immunotherapy, represented by immune checkpoint inhibitors, especially PD1/PD-L1, is a promising therapeutic approach that has been developed in recent years. It has shown significant efficacy in some patients with advanced colorectal cancer [45]. To explore the relationship between EphA2 expression and immune cell infiltration in colorectal cancer, we analyzed the RNA-seq expression profile data of TCGA as well as clinically relevant information and found that EphA2 expression was significantly and positively correlated with macrophage, neutrophil and myeloid dendritic cell infiltration (Figure 6A, 6B). We further analyzed the correlation between EphA2 expression and immune checkpoint expression using TCGA expression profile data and found no significant correlation between EphA2 and common immune checkpoints such as CD274, CTLA4, TIGIT, PDCD1, etc. (Figure 6C). These results suggest that EphA2 may influence colorectal carcinogenesis and progression through the infiltration of some immune cells.

## DISCUSSION

EphA2 belongs to the Eph receptor family, which is the largest family of receptor tyrosine kinases. Eph receptors can be classified into EphA and EphB types depending on their sequence homology, structure, affinity for the ligand to which they bind, and distribution. Humans express nine EphA receptors and five EphB receptors [46]. EphA2 is highly expressed in many tumours and is of prognostic importance. For example, EphA2 may promote the progression of squamous head and neck cancer and is expected to be a prognostic indicator [47]. In non-small cell lung cancer, EphA2 reduces progression-free survival and overall survival of patients [48]. In gastric cancer, EphA2 can promote tumor cell progression by activating the Wnt signaling pathway [9]. In this study, we found that EphA2 was abnormally highly expressed in colorectal cancer and that patients with high expression had a worse prognosis. In addition, we also found that EphA2 can undergo phase separation and a positive correlation with the expression of ferroptosis-related genes and immune cell infiltration, but whether other members of the Eph receptor family have similar phenomena in colorectal cancer requires further evidence.

The phase separation is involved in the regulation of cellular metabolism, signal transduction, gene expression and protein homeostasis. It is important for the maintenance of homeostasis in the organism. The relationship between phase separation and cancer has recently attracted much attention [49–51]. In colorectal cancer, SENP1 inhibits RNF168 phase separation, which in turn promotes DNA damage repair and drug resistance in colorectal cancer [34]. NUP98 fusion oncoprotein is a driver of childhood leukaemia. Most NUP98 fusion proteins contain intrinsically disordered regions that are susceptible to liquid-liquid phase separation (LLPS) *in vitro*. Phase separation of NUP98 fusion oncoproteins is required to mediate leukemic transformation [52]. Phosphatidic acid-binding lncRNA SNHG9 promotes LATS1 liquid-liquid phase separation and facilitates oncogenic YAP signaling [53]. In this study, we found that EphA2 has a distinct internal disordered region and that EphA2 can form puncta on cell membranes, further demonstrating that these puncta are formed by LLPS, but further experiments are needed to prove whether EphA2 exerts a pro-cancer function through its phase separation properties. In addition, EphA2 acts as a transmembrane receptor that transmits a variety of extracellular signals into the cell. Whether EphA2 phase-separated condensate can activate downstream signaling pathways by condensing other signaling molecules needs to be further explored. Most previous studies have focused on nuclear proteins such as transcription factors having phase separation [40, 54], and in our study the membrane receptor EphA2 was found to have phase separation. Recently there is also a growing number of other membrane molecules that can function by phase separation, such as DIAPH3 condensates formed by liquid-liquid phase separation act as a regulatory hub for stress-induced actin cytoskeleton remodeling [55]. CD3ε, a component of TCR, can intrinsically form phase separation with Lck through ionic interactions. The condensate structure significantly promotes Lck-mediated CD3 phosphorylation to yield the amplification of TCR signaling [56].

Ferroptosis is a novel mode of cell death that has been shown to be involved in the development of many cancers and to increase the susceptibility of cancers to radiotherapy, providing a new theoretical basis for the prevention and treatment of cancer. For example, in hepatocellular carcinoma donafenib and GSK-J4 synergistically induce ferroptosis in tumor cells through upregulation of HMOX1 expression [57]. ASCL1 can promote progression of castration-resistant prostate cancer to neurosecretory prostate cancer by mediating ferroptosis resistance [58]. In gastric cancer cells, the HNF4A-BAP31-VDAC1 axis synchronously regulates tumor cell proliferation and ferroptosis [59]. The occurrence of ferroptosis is regulated by several genes, such as GPX4, CDKN1A, SLC1A5, and ACSL4, etc. In this study, we found that EphA2 expression showed significant correlation with several ferroptosis-related genes, including ALOX15, ATL1, CARS1, HSPA5, SLC7A11 and SLC1A5, etc., [42], suggesting that EphA2 may influence tumour progression by regulating iron death in colorectal cancer.

In recent years, the emergence of tumor immunotherapy has changed the traditional pattern of tumor treatment. It is one of the most promising research directions in the field of tumour therapy, and has been widely used as the fourth tumour treatment in clinical practice [60]. Tumour immunotherapy is mainly divided into immune checkpoint blockades (ICBs) and chimeric antigen receptor T cell immunotherapy. In this study, we found that EphA2 expression correlated with infiltration of a variety of immune cells, such as neutrophils, myeloid dendritic cells and macrophage cells, but EphA2 expression did not correlate significantly with common immune checkpoints such as PDCD1, CTLA4, and CD274, etc., These results suggest that EphA2 may regulate the development of colorectal cancer by influencing the infiltration of immune cells.
